# Predicting lymphovascular invasion in rectal cancer: evaluating the performance of golden-angle radial sparse parallel MRI for rectal perfusion assessment

**DOI:** 10.1038/s41598-023-35763-8

**Published:** 2023-05-25

**Authors:** Yingying Fan, Meining Chen, Hongyun Huang, Mi Zhou

**Affiliations:** 1 Department of Radiology, Sichuan Provincial People’s Hospital, University of Electronic Science and Technology of China, No.32, West Second Section of First Ring Road, Qingyang District, Chengdu, 610072 People’s Republic of China; 2 MR Scientific Marketing, Siemens Healthineers, Shanghai, China

**Keywords:** Gastrointestinal cancer, Cancer imaging

## Abstract

This study aims to determine whether the dual-parameter approach combined with either time-resolved angiography with stochastic trajectories (TWIST) or golden-angle radial sparse parallel (GRASP) and diffusion-weighted imaging (DWI) has superior diagnostic performance in predicting pathological lymphovascular invasion (pLVI) rectal cancer when compared with traditional single-parameter evaluations using DWI alone. Patients with pathologically confirmed rectal cancer were enrolled. Perfusion (influx forward volume transfer constant [Ktrans] and rate constant [Kep]) and apparent diffusion coefficient (ADC) were measured by two researchers. For both sequences, areas under receiver operating characteristic (ROCs) to predict pLVI-positive rectal cancer were compared. A total of 179 patients were enrolled in our study. A combined analysis of ADC and perfusion parameters (Ktrans) acquired with GRASP yielded a higher diagnostic performance compared with diffusion parameters alone (area under the curve, 0.91 ± 0.03 vs. 0.71 ± 0.06, P < 0.001); However, ADC with GRASP-acquired Kep and ADC with TWIST-acquired perfusion parameters (Ktrans or Kep) did not offer any additional benefit. The Ktrans of the GRASP technique improved the diagnostic performance of multiparametric MRI to predict rectal cancers with pLVI-positive. In contrast, TWIST did not achieve this effect.

## Introduction

Colorectal cancer is one of the leading causes of cancer morbidity and mortality worldwide^1^. Several factors determine its prognosis, including differentiation grade, T stage, and lymphovascular invasion (LVI)^2–4^. LVI, defined as the infiltration of tumor cells into lymphatic or blood vessels at the periphery of invasive carcinoma^5,6^, has been widely recognized as a negative prognostic factor in rectal cancer^7^. Even so, LVI has not been included as an important parameter to consider before adjuvant chemotherapy in the National Comprehensive Cancer Network guidelines due to difficulty in determining it before adjuvant chemotherapy; it can only be postoperatively diagnosed by histopathology^8^. Thus, more evidence is needed to warrant the application of LVI in clinical decision-making.

MR imaging has been used as an important technique for rectal cancer staging and estimates tumor vascular permeability on the basis of pharmacokinetic modeling of the tumor gadolinium concentration with respect to the plasma compartment^9^. Diffusion-weighted imaging (DWI) and dynamic contrast-enhanced magnetic resonance imaging (DCE-MRI) are functional MRI imaging techniques that yield qualitative and quantitative information and provide unique insights regarding tumor cellularity, the integrity of cell membranes, and microcirculation^10,11^. It has been shown that quantitative DCE-MRI parameters and apparent diffusion coefficient (ADC) values correlate closely with histological grade^12,13^, response to neoadjuvant chemoradiotherapy (CRT) and tumor prognostic factors^14^.

However, the view-sharing time-resolved angiography with stochastic trajectories (TWIST) technique, due to its high acceleration, is prone to motion artifacts caused by bowel movement or patient motion, which may reduce the diagnostic accuracy of the examination^15^. In addition, its accuracy is also compromised by a mismatch in timing between the administration of the contrast agent and the acquisition of the image as well as the typically rather low spatial resolution^15,16^.

Recently, golden-angle radial sparse parallel MRI (GRASP) has been proposed for rapid free-breathing dynamic MRI^16^. To improve temporal resolution, a motion- insensitive Golden Angle, the stack-of-stars acquisition, is combined with a compressed sensing reconstruction and is well suited for free-breathing DCE-MRI^15^. It has been applied to several DCE-MRI studies, including liver, prostate, breast, bladder, kidney, and rectum^15–22^. Previous studies have shown that GRASP perfusion could yield equivalent image quality and fewer motion artifacts than conventional DCE for rectal cancer imaging^15,22^. Although all the above-mentioned studies have achieved satisfactory results, to our knowledge, the diagnostic performance of quantitative parameters of GRASP for LVI-positive rectal cancer has not yet been well determined. Moreover, previous studies^23–25^ mainly focused on preoperative therapy response, staging, and prognostic assessment in rectal cancer by DCE-MRI or DWI. Few studies have been carried out focusing on the diagnostic performance of LVI positivity from quantitative DCE-MRI and DWI in rectal cancer. Consequently, our objective was to assess the diagnostic performance of a dual-parameter approach that combines either TWIST or GRASP with established DWI for predicting rectal cancer with LVI in comparison to a traditional single-parameter evaluation based on DWI alone.

## Methods

### Ethics statement

This retrospective study was approved by the Institutional Review Board named Sichuan Provincial People's Hospital, University of Electronic Science and Technology of China. All research methods were conducted in accordance with the relevant guidelines and regulations. The need for written informed consent was waived by the institutional review board due to the retrospective design of the study.

### Patient inclusion

Consecutive patients with nonmucinous rectal adenocarcinoma diagnosed by endoscopy-guided biopsy between December 2020 and October 2022 were included. The inclusion criteria were patients who had pathologically confirmed rectal cancer after surgical resection, patients with complete MRI images, and patients who had not received neoadjuvant chemoradiotherapy at the time of the MRI scan. The exclusion criteria were as follows: (A) general MRI contraindications such as severely restricted kidney function, certain pacemakers, metal implants, claustrophobia, pregnancy; (B) there was unresectable or metastatic diseases; (C) mucinous cystadenoma was in evidence. Patients were randomized into two groups, GRASP and TWIST.

### MRI acquisition

All MRI scans were acquired using 3.0 T and 1.5 T MR systems (MAGNETOM Vida and MAGNETOM Aera; Siemens Healthineers, Shanghai, China). GRASP acquisitions were only performed using the Vida system, and TWIST acquisitions were only performed using the Aera system by employing a 30-channel coil setup (18-channel body coil and 12 channels from the spine coil) for Vida and 18-channel coil setup (6-channel body coil and 12 channels from the spine coil) for Aera. Patients were placed head first and supine on the table. Bowel cleansing with an enema was performed 50 min before the examination. Patients were then administered 20 mg of scopolamine butyl bromide (Buscopan, Boehringer Ingelheim) intramuscularly 30 min before the scan to minimize bowel motion.

The conventional MRI protocol included sagittal, axial (perpendicular to the long axis of the rectum), oblique coronal T_2_-weighted images without fat saturation and DWI (perpendicular to the long axis of the rectum). The acquisition parameters for T_2_-weighted images were as follows: TR/TE, 4590/73; field of view, 220 × 220 mm^2^; matrix size, 256 × 512; section thickness, 3.5 mm; and intersection gap, 0.7 mm. Axial DW images of the pelvis were obtained with the following parameters: 4600/59; several signals acquired, eight; field of view, 360 mm^2^; section thickness, 4.5 mm; and b values, 50 and 1000 s/mm^2^^26–28^.

GRASP or TWIST perfusion was measured after administering body weight-adapted intravenous gadopentetate dimeglumine (0.1 mmol/kg BW, Dotarem; Guerbet, Paris, France) at a rate of 2 mL/s. Protocol parameters for TWIST and GRASP acquisition are detailed in Table 1.

**Table 1:** Parameters for TWIST and GRASP.

	TWIST	GRASP
TR	3.6 ms	3.5 ms
TE	1.44 ms	1.64 ms
Slice thickness	3.6 mm	3 mm
Matrix	192 × 130	256 × 256
FOV	320 × 320 mm^2^	273 × 394 mm^2^
FA	12 degrees	12 degrees
Temporal resolution	4.88 s	3.45 s

GRASP, golden-angle radial sparse parallel MRI; TWIST, time-resolved angiography with interleaved stochastic trajectories; TR, repetition time; TE, echo time; FOV, field of view; FA, flip angle.

### Image analysis

Image processing was performed by using a commercially available software application (Syngo. via VB30, MR Prostate, and MR Tissue4D; Siemens Healthineers, Shanghai, China). The TOFTS model was used to calculate quantitative pharmacokinetic model parameters, including the influx forward volume transfer constant (Ktrans, /min) and rate constant (Kep, /min).

**Figure 1:**
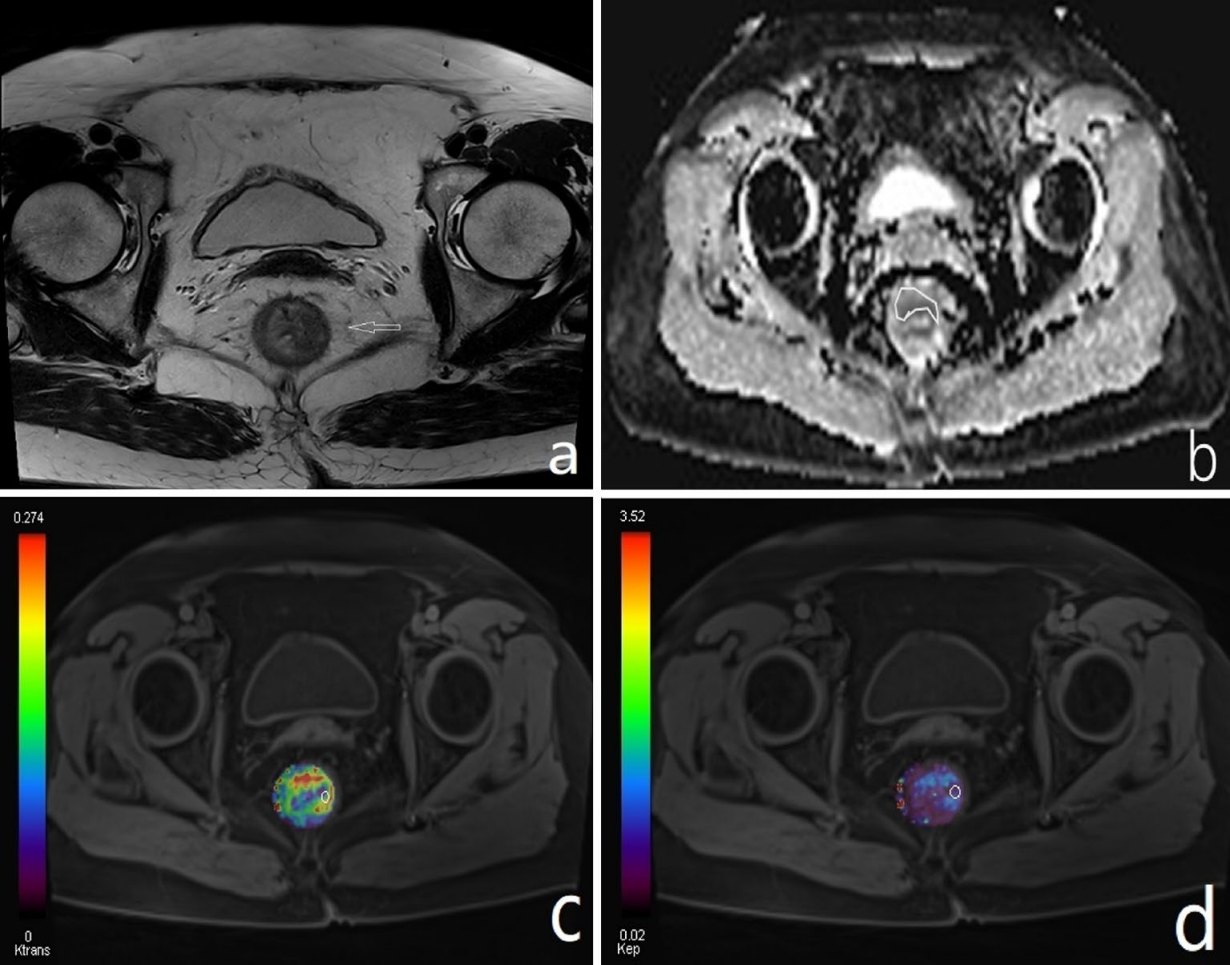
(**a**–**d**) Showing the T2WI, ADC map, Ktrans, and Kep of GRASP, respectively.

**Figure 2:**
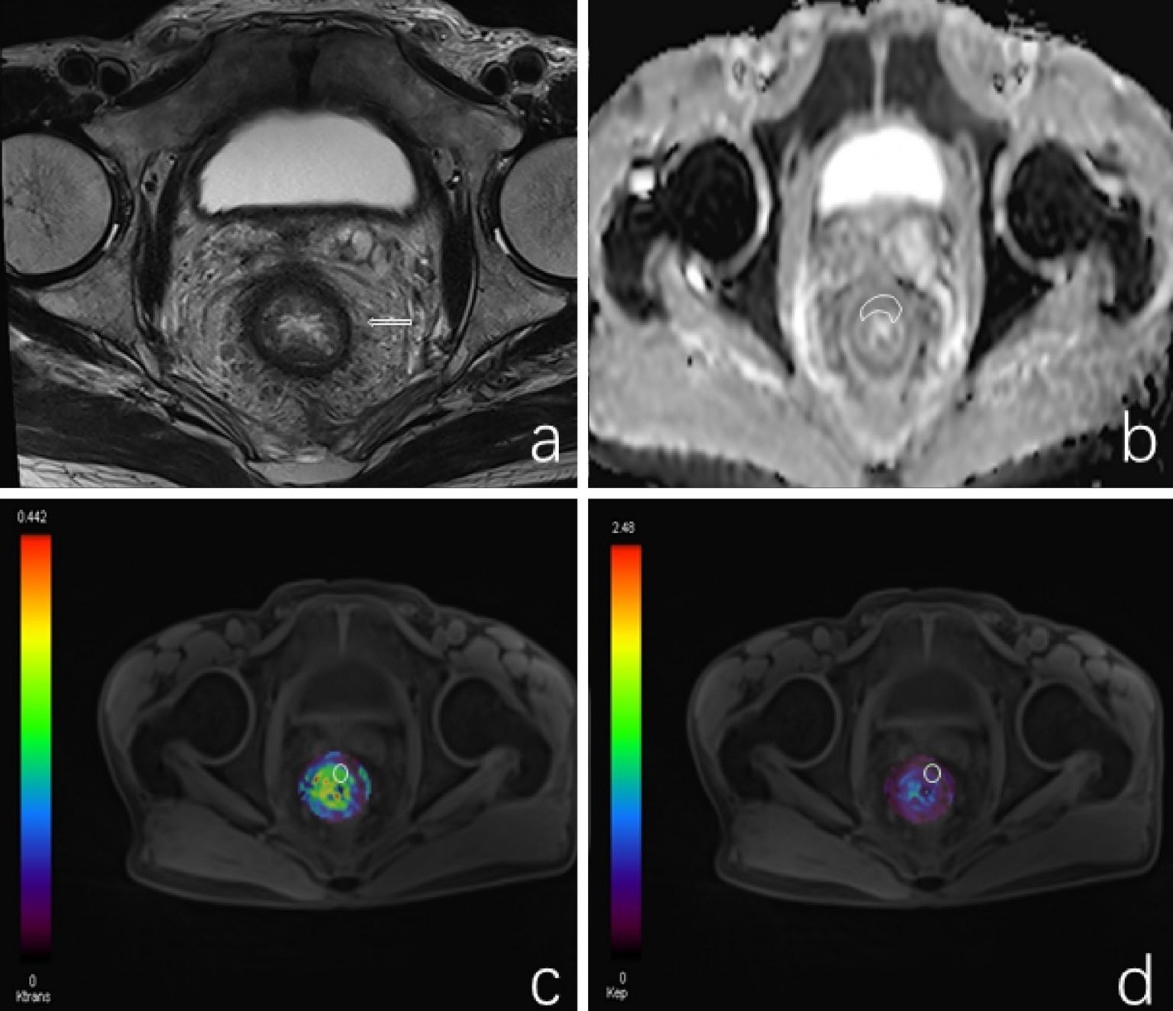
(**a**–**d**) Showing the T2WI, ADC map, Ktrans, and Kep of TWIST, respectively.

Ktrans and Kep measurements were achieved by a circle tool to delineate the ROI on perfusion maps with the largest three layers of tumor lesions (carefully avoiding necrosis or cystic areas). In this study, two experienced radiologists (with 6 and 10 years of experience in rectal imaging) performed this task blind to the patient’s clinical and pathological information, but they were aware that the patients were rectal cancer patients. The radiologists reviewed the T_2_WI and DWI images and determined the location of the tumor. The final Ktrans and Kep values corresponded to the mean values obtained by drawing three different levels of ROI (with areas no less than 1 cm^2^)^29^ and taking the average. The Ktrans and Kep values were averaged between the two radiologists for further analysis (Figs. 1, 2).

### The ADC value

ADC measurement was achieved by drawing the freehand ROI on the central slice of the tumor (b = 1000 s/mm^2^); this task was performed by two experienced radiologists (with 6 and 10 years of experience in rectal imaging). The ROIs were drawn to cover the entire tumor area with sufficient size and then copied to the ADC maps. In addition, areas of necrosis, vessels, and cysts, as identified on T_2_-weighted images, were avoided to minimize bias. The final ADC values corresponded to the mean values obtained by drawing three randomized ROIs in different tumor areas on three independent tumor-containing slices. The ADC values were averaged between the two radiologists for further analysis (Figs. 1, 2).

### Pathological examinations

The presence of microscopic LVI was confirmed by a pathologist with 6 years of experience in pathology. LVI was assessed on hematoxylin and eosin-stained sections and was defined as carcinoma cells in a definite endothelial-lined space in the peritumoral rectum surrounding the invasive carcinoma. LVIs were classified into four grades: ly/v 0 (no LVI), ly/v 1 (minimal LVI), ly/v 2 (moderate LVI), and ly/v 3 (marked LVI). We divided LVIs into LVI-positive (ly/v 1–3) and LVI-negative groups (ly/v 0)^30^.

### Statistical analysis

The statistical analyses were performed using SPSS version 26 (IBM Corporation) and MedCalc (Version 16.8). Inter-observer variability of the continuous variables was assessed by using intraclass correlation coefficients (ICCs). The ICC was classified into poor (ICC < 0.2), fair (0.21–0.4), moderate (0.41–0.60), good (0.61–0.80), and excellent (0.81–1.00) agreement^31^. All quantitative ADC, Ktrans, and Kep parameters are presented as the mean ± standard deviation with range. The independent samples t test and chi-square test were used to compare the clinical information and quantitative parameters of the two groups. Statistical analyses were performed to determine whether a combined diffusion and perfusion parameter assessment could achieve higher diagnostic accuracy than a single-parameter evaluation based solely on diffusion measurements. The following parameters were evaluated: ADC combined with Ktrans and ADC combined with Kep versus ADC alone. To determine the optimal cutoffs for each parameter, receiver operating characteristic (ROC) curves were used to determine the points that maximized Youden’s index. Youden’s index was calculated as specificity + sensitivity−1^32^. The DeLong test was used to compare the ROC curves. To adjust for multiple testing and to control the type I error in our study, we performed a Bonferroni correction of the significance level of the individual test with the following formula: p*$\frac{\alpha }{m}$^33^, where p * is the adjusted significance level, $\alpha$ is the critical P value, and $m$ is the number of comparisons. A two-sided p value < 0.05 represented statistical significance.

$${\rm Accuracy\, was \, defined \, as}: \frac{{true \;positive + \left( {true \;negative} \right)}}{{true\; positive + true \;negative + false\, positive + \left( {false\; negative} \right)}}$$

## Results

### Patient characteristics

**Figure 3:**
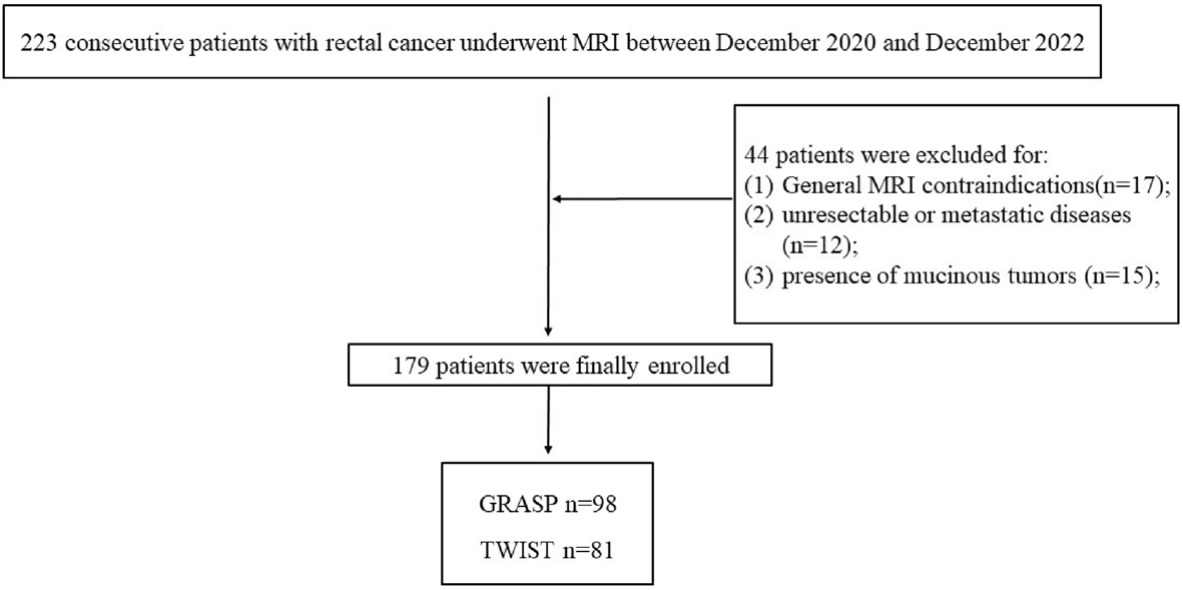
Flow diagram showing the inclusion and exclusion criteria for the study.

Based on the clinical history and physical examination results, 223 patients with clinically suspected rectal cancer were enrolled. Finally, 179 patients (98 examined by the GRASP technique and 81 examined by the TWIST technique) who underwent MRI were included in this study Fig. 3. There was no statistically significant difference between the two groups in terms of sex, age, or tumor location (P > 0.05) (Table 2).

**Table 2:** Clinical information of the two groups of patients.

	TWIST (*n* = 81)	GRASP (*n* = 98)	*P* value
Gender, *n *(%)			0.549
Male	65 (80.2)	75 (76.5)	
Female	16 (19.8)	23 (23.5)	
Age(years)	63.25 ± 10.85	61.24 ± 10.43	0.211
Tumor location, *n *(%)			0.938
High	27 (33.3)	32 (32.7)	
Medium	36 (44.4)	42 (42.9)	
Low	18 (22.2)	24 (24.5)	

Note: GRASP, golden-angle radial sparse parallel; TWIST, view-sharing time-resolved angiography with stochastic trajectories technique.

The test level for the P value was 0.05.

### Interobserver agreement between the two groups

Interobserver agreement was excellent for Ktrans (ICC, 0.954; 95% CI: 0.774–0.984), Kep (ICC, 0.917; 95% CI: 0.820–0.962), and ADC (ICC, 0.911; 95% CI: 0.693–0.966) in the TWIST group; Interobserver agreement was excellent for Ktrans (ICC, 0.908; 95% CI: 0.399–0.971), Kep (ICC, 0.922; 95% CI: 0.699–0.970), and ADC (ICC, 0.909; 95% CI: 0.221–0.974) in the GRASP group.

### Assessment of the diagnostic performance of DCE MRI and DWI

The ADC value of pLVI-positive rectal cancer was significantly lower than that of pLVI-negative rectal cancer for the TWIST group (0.93 ± 0.18 vs. 1.15 ± 0.13, P < 0.001) and the GRASP group (0.98 ± 0.13 vs. 1.10 ± 0.16, P < 0.001). The Ktrans and Kep of pLVI-positive rectal cancer were significantly higher than those of pLVI-negative rectal cancer for both groups (all P < 0.001) as shown in Table 3. Table 4 shows the diagnostic performance of different models for the discrimination of pLVI-positive rectal cancer. Ktrans (cutoff value, 0.67/min) showed higher accuracies to detect pLVI-positive rectal cancer than ADC (cutoff value, 0.95 × 10^–3^ s/mm^2^) or Kep (cutoff value, 0.93/min) in the GRASP group.

**Table 3:** Quantitative results of predicting pLVI-positive from pLVI-negative rectal cancer.

TWIST (*n* = 81)	pLVI-negative (*n* = 58)	pLVI-positive (*n* = 23)	*P* value
ADC-T	1.15 ± 0.13	0.93 ± 0.18	< 0.001
Ktrans-T	0.58 ± 0.16	0.94 ± 0.29	< 0.001
Kep-T	0.82 ± 0.18	1.00 ± 0.28	0.001

GRASP (*n* = 98)	pLVI-negative (*n* = 74)	pLVI-positive (*n* = 24)	*P* value
ADC-G	1.10 ± 0.16	0.98 ± 0.13	0.002
Ktrans-G	0.61 ± 0.18	0.86 ± 0.15	< 0.001
Kep-G	0.84 ± 0.25	1.03 ± 0.23	0.002

Note. Data are the mean ± standard deviation unless otherwise indicated. P values less than 0.05 were considered to indicate statistical significance.

ADC, apparent diffusion coefficient; GRASP, golden-angle radial sparse parallel; Ktrans, influx forward volume transfer constant; Kep, rate constant; VIBE, volumetric interpolated breath-hold examination; pLVI, pathological lymphovascular invasion.

**Table 4:** Performance of models for predicting pLVI-positive rectal cancer.

Group	ADC			Ktrans			Kep		
	Accuracy	AUC	Cutoff	Accuracy	AUC	Cutoff	Accuracy	AUC	Cutoff
TWIST (*n* = 81)	85 (69/81)	0.80 ± 0.06	1.05	90 (73/81)	0.87 ± 0.06	0.84	72 (58/81)	0.70 ± 0.07	0.92
GRASP (*n* = 98)	65 (66/98)	0.71 ± 0.06	0.95	68 (67/98)	0.85 ± 0.04	0.67	61 (60/98)	0.68 ± 0.06	0.93

Group	ADC with Ktrans			ADC with Kep			*P value*		
	Accuracy	AUC	Cutoff	Accuracy	AUC	Cutoff	ADC vs. ADC with Ktrans	ADC vs. ADC with Kep	
TWIST (*n* = 81)	85 (69/81)	0.91 ± 0.04	0.24	90 (73/81)	0.82 ± 0.06	0.43	0.076	0.511	
GRASP (*n* = 98)	87 (85/98)	0.91 ± 0.03	0.38	80 (78/98)	0.79 ± 0.06	0.32	< 0.001	0.061	

Note: Data in parentheses are numerators/denominators. Areas under the curve are ± standard error. Perfusion parameters derived from the TWIST and GRASP MRI study participant groups are shown. P < 0.025 was considered to indicate statistical significance.

ADC, apparent diffusion coefficient; AUC, area under the curve; GRASP, golden-angle radial sparse parallel; Kep, rate constant; Ktrans, influx forward volume transfer constant; ROC, receiver operating characteristic; TWIST, view-sharing time-resolved angiography with stochastic trajectories technique; pLVI, pathological lymphovascular invasion.

### Assessment of single- versus dual-parameter models

**Figure 4:**
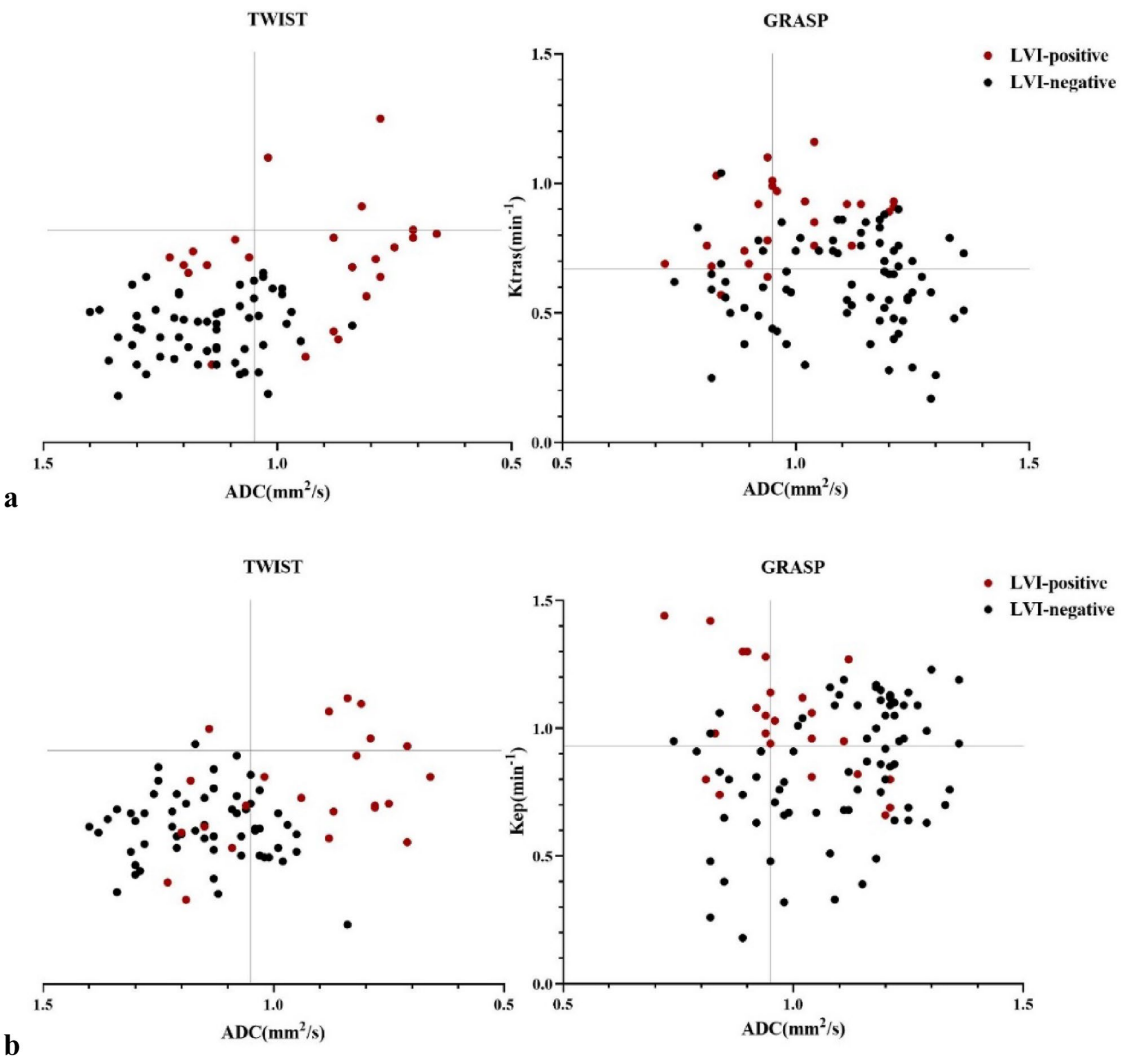
Quantitative assessment of combined diffusion MRI and dynamic contrast agent–enhanced MRI. Scatterplots display the data pairs of apparent diffusion coefficients (ADCs) with (**a**) influx forward volume transfer constant and (**b**) ADC with rate constant values. ADC values are on the x-axis, and the perfusion parameters are on the y-axis. Horizontal and vertical lines represent the dual-parameter cutoff levels.

Combining ADC with perfusion parameters using a binary logistic regression approach. According to the dual-parameter analysis, the combination of ADC cutoff levels and GRASP-based Ktrans offered significantly better diagnostic performance than a single-factor evaluation based solely on ADC cutoff levels for the discrimination of pLVI-positive rectal cancer (ADC with Ktrans vs. ADC, P < 0.001). In terms of single-parameter evaluation, Ktrans provided the best results. In the combined analysis of ADC and perfusion cutoff levels based on TWIST, this statistical level of improvement was not observed. Table 4. Figure 4 shows that when the cutoff levels are based on GRASP-derived perfusion parameters combined with ADC, pLVI-positive rectal cancer can be distinguished more effectively (fewer overlaps) compared to the cutoff levels based on TWIST-derived perfusion parameters combined with ADC.

## Discussion

We found that a dual-parameter approach incorporating Ktrans of GRASP and established DWI allows better diagnosis of pLVI in rectal cancer than a traditional single-parameter assessment based solely on DWI. The Ktrans and Kep of pLVI-positive rectal cancer were significantly higher than those of pLVI-negative rectal cancer (P < 0.05).

Igarashi et al.^30^ reported that the tumor ADC value is a significant predictor of LVI in breast cancer. In our study, the ADC of pLVI-positive rectal cancer was significantly lower than that of pLVI-negative rectal cancer (p < 0.05), which was consistent with previous studies^34,35^. This results from the fact that with the increase in the degree of cancerous behavior of malignant tumor, the proliferation of tumor cells significantly accelerates. The ratio of the nucleus and the cell density per unit volume increases, leading to a decrease in the extracellular space distance and free diffusion of water molecules, limitation of distribution, and in turn, a lower ADC value^12^. Choi et al.^6^ found no significant correlation for pLVI with either the minimum, maximum, or mean ADC values (P > 0.05). The reason for the discordance between our results and those of others may be attributed to several factors, including the use of different ADC measurements, inability to capture tumor heterogeneity when calculating mean diffusion parameters obtained from single-section regions or multiple small ROIs, and the choice of different b-values^35^.

Ktrans reflects the ability of the contrast agent to be transported from the blood vessels to the interstitial space, the higher the degree of malignancy, the more capillaries there are, leading to a higher Ktrans value^5^. Similarly, a higher Kep value represents greater blood return to the vasculature. Therefore, a higher Kep value indicates more leakage of the contrast medium. Our study found that Ktrans and Kep of pLVI-positive rectal cancer were significantly higher than those of pLVI-negative rectal cancer (P < 0.05), which was consistent with previous studies^7^. This is because LVI strongly correlates with a high peritumoral lymphovascular density and more aggressive neovascularization, and these alterations induce differences in the volume and flow of blood in the tumor microcirculatory environment^5,36–38^. However, according to Lai et al.^8^, there was no significant difference in Ktrans between pLVI-positive and pLVI-negative groups (P > 0.05) in breast cancer. The reason could be due to the large sample size of our study and the complexity of the underlying pathophysiology of heterogeneous rectal cancer. Our study primarily focused on evaluating the diagnostic performance of multiparametric MRI to predict rectal cancers with pLVI in general, rather than differentiating between the three distinct states of positive vascular infiltration: lymphatic infiltration, vascular infiltration, and both infiltration. These different states may have varying impacts on prognosis and diffusion and perfusion parameters. The current literature on this topic is limited, and further studies are needed to determine the specific effects of each type of infiltration on diffusion and perfusion parameters, such as Ktrans and kep.

Quantitative DCE-MRI parameters and ADC values were reported to be closely correlated with clinical and histological grade, response to neoadjuvant chemoradiotherapy (CRT), and prognostic factors of various tumors^14,39,40^. There are some limitations of conventional DCE-MRI, however. First, the temporal resolution is approximately 5–18 s per phase. Second, the acquisitions require breath holds, which can be challenging in some patients and can restrict spatiotemporal resolution and volumetric coverage in dynamic imaging acquisitions^41–45^. In our study, we utilized radial acquisition with GRASP, which increased spatial and temporal resolutions compared to TWIST. According to the literature^46,47^, the higher the temporal resolution, the more accurate the semiquantitative and quantitative parameters obtained during the DCE scan will be. The GRASP sequence provides an enhanced spatial and temporal resolution, which is particularly beneficial for accurately assessing perfusion parameters, during DCE scans^15^. Our results demonstrated that the integration of ADC values with Ktrans acquired from the GRASP sequence yielded a higher diagnostic performance compared to diffusion parameters alone. This finding suggests that the enhanced spatial and temporal resolution of the GRASP sequence contributes to better discrimination of pLVI-positive rectal cancer.

Winkle et al.^48^ revealed that GRASP had been shown to improve the diagnostic accuracy of multiparametric MRI examinations of the prostate when incorporated into a dual-parameter model that included diffusion and perfusion characteristics. Ao et al.^45^ reported that the Ktrans and ADC values were independent predictors of extramural venous invasion in rectal cancer. According to the study by Oberholzer et al.^49^, MR perfusion may serve as a complementary biomarker to ADC values to assess tumor characteristics associated with the effectiveness of chemoradiation before treatment initiation. We found that the dual-parameter analyses, which combined ADC values with the Ktrans from GRASP, provided a better diagnosis of pLVI-positive rectal cancers than the single-factor analysis of ADC. GRASP was the only technique used to calculate perfusion maps, resulting in a statistically significant difference in tumor detection. Thus, it may be hypothesized that the combined increased spatial and temporal resolution of the former acquisition method benefits the discrimination of pLVI-positive rectal cancer.

Our study had limitations. First, the TWIST and GRASP groups were scanned in MR scanners with different field strengths, which may affect the measurement of perfusion and diffusion parameters. Second, it is essential to note that this is a single institutional study without a validation cohort, so future studies are required to determine whether our results can be replicated in other medical institutions. Third, the retrospective design, which may predispose to selection bias. Finally, the ROI outlines the individual tumor level rather than the entire tumor, which may better reflect the perfusion parameters of the tumors (Supplementary Information).

## Conclusions

Despite these limitations, our results indicate that Ktrans acquired from GRASP techniques significantly enhanced the diagnostic performance of multiparametric MR examinations in predicting rectal cancer with LVI-positive when integrated into a dual-parameter model by incorporating diffusion and perfusion characteristics. In contrast, Kep acquired from GRASP and TWIST perfusion parameters did not exhibit this effect.

## Supplementary Information

Supplementary Information.

## Data Availability

The datasets used and/or analysed during the current study available from the corresponding author on reasonable request.

## References

[1] Wu CC, Lee RC, Chang CY (2013). Prediction of lymphovascular invasion in rectal cancer by preoperative CT. AJR Am. J. Roentgenol..

[2] Yang YS, Qiu YJ, Zheng GH (2021). High resolution MRI-based radiomic nomogram in predicting perineural invasion in rectal cancer. Cancer Imaging.

[3] Yang YS, Feng F, Qiu YJ, Zheng GH, Ge YQ, Wang YT (2021). High-resolution MRI-based radiomics analysis to predict lymph node metastasis and tumor deposits respectively in rectal cancer. Abdom. Radiol. (N.Y.).

[4] Bown EJ, Lloyd GM, Boyle KM, Miller AS (2014). Rectal cancer: Prognostic indicators of long-term outcome in patients considered for surgery. Int. J. Colorectal Dis..

[5] Zhu Y, Zhou Y, Zhang W (2021). Value of quantitative dynamic contrast-enhanced and diffusion-weighted magnetic resonance imaging in predicting extramural venous invasion in locally advanced gastric cancer and prognostic significance. Quant. Imaging Med. Surg..

[6] Choi BB (2021). Dynamic contrast enhanced-MRI and diffusion-weighted image as predictors of lymphovascular invasion in node-negative invasive breast cancer. World J. Surg. Oncol..

[7] Yu J, Xu Q, Huang DY (2017). Prognostic aspects of dynamic contrast-enhanced magnetic resonance imaging in synchronous distant metastatic rectal cancer. Eur. Radiol..

[8] Lai T, Chen X, Yang Z (2022). Quantitative parameters of dynamic contrast-enhanced magnetic resonance imaging to predict lymphovascular invasion and survival outcome in breast cancer. Cancer Imaging.

[9] Tofts PS (1997). Modeling tracer kinetics in dynamic Gd-DTPA MR imaging. J. Magn. Reson. Imaging.

[10] DeVries AF, Kremser C, Hein PA (2003). Tumor microcirculation and diffusion predict therapy outcome for primary rectal carcinoma. Int. J. Radiat. Oncol. Biol. Phys..

[11] Engin G, Sharifov R, Güral Z (2012). Can diffusion-weighted MRI determine complete responders after neoadjuvant chemoradiation for locally advanced rectal cancer?. Diagn. Intervent. Radiol. (Ankara, Turkey).

[12] Curvo-Semedo L, Lambregts DM, Maas M, Beets GL, Caseiro-Alves F, Beets-Tan RG (2012). Diffusion-weighted MRI in rectal cancer: Apparent diffusion coefficient as a potential noninvasive marker of tumor aggressiveness. J. Magn. Reson. Imaging.

[13] Li M, Xu X, Xia K (2021). Comparison of diagnostic performance between perfusion-related intravoxel incoherent motion DWI and dynamic contrast-enhanced MRI in rectal cancer. Comput. Math. Methods Med..

[14] Gürses B, Böge M, Altınmakas E, Balık E (2019). Multiparametric MRI in rectal cancer. Diagn. Intervent. Radiol. (Ankara, Turkey).

[15] Attenberger UI, Liu J, Riffel P (2017). Quantitative perfusion analysis of the rectum using golden-angle radial sparse parallel magnetic resonance imaging: Initial experience and comparison to time-resolved angiography with interleaved stochastic trajectories. Invest. Radiol..

[16] Riffel P, Zoellner FG, Budjan J (2016). "One-stop shop": Free-breathing dynamic contrast-enhanced magnetic resonance imaging of the kidney using iterative reconstruction and continuous golden-angle radial sampling. Invest. Radiol..

[17] Chandarana H, Feng L, Block TK (2013). Free-breathing contrast-enhanced multiphase MRI of the liver using a combination of compressed sensing, parallel imaging, and golden-angle radial sampling. Invest. Radiol..

[18] Chandarana H, Block TK, Ream J (2015). Estimating liver perfusion from free-breathing continuously acquired dynamic gadolinium-ethoxybenzyl-diethylenetriamine pentaacetic acid-enhanced acquisition with compressed sensing reconstruction. Invest. Radiol..

[19] Rosenkrantz AB, Geppert C, Grimm R (2015). Dynamic contrast-enhanced MRI of the prostate with high spatiotemporal resolution using compressed sensing, parallel imaging, and continuous golden-angle radial sampling: Preliminary experience. J. Magn. Reson. Imaging.

[20] Kim SG, Feng L, Grimm R (2016). Influence of temporal regularization and radial undersampling factor on compressed sensing reconstruction in dynamic contrast enhanced MRI of the breast. J. Magn. Reson. Imaging.

[21] Parikh N, Ream JM, Zhang HC, Block KT, Chandarana H, Rosenkrantz AB (2016). Performance of simultaneous high temporal resolution quantitative perfusion imaging of bladder tumors and conventional multi-phase urography using a novel free-breathing continuously acquired radial compressed-sensing MRI sequence. Magn. Reson. Imaging.

[22] Li Y, Xia C, Peng W (2020). Dynamic contrast-enhanced MR imaging of rectal cancer using a golden-angle radial stack-of-stars VIBE sequence: Comparison with conventional contrast-enhanced 3D VIBE sequence. Abdom. Radiol. (N. Y.).

[23] Prampolini F, Taschini S, Pecchi A (2020). Magnetic resonance imaging performed before and after preoperative chemoradiotherapy in rectal cancer: Predictive factors of recurrence and prognostic significance of MR-detected extramural venous invasion. Abdom. Radiol. (N. Y.).

[24] Wu LF, Rao SX, Xu PJ (2019). Pre-TACE kurtosis of ADC(total) derived from histogram analysis for diffusion-weighted imaging is the best independent predictor of prognosis in hepatocellular carcinoma. Eur. Radiol..

[25] Granata V, Grassi R, Fusco R (2020). Current status on response to treatment in locally advanced rectal cancer: What the radiologist should know. Eur. Rev. Med. Pharmacol. Sci..

[26] Xia CC, Liu X, Peng WL (2016). Readout-segmented echo-planar imaging improves the image quality of diffusion-weighted MR imaging in rectal cancer: Comparison with single-shot echo-planar diffusion-weighted sequences. Eur. J. Radiol..

[27] Lambregts DMJ, van Heeswijk MM, Delli Pizzi A (2017). Diffusion-weighted MRI to assess response to chemoradiotherapy in rectal cancer: Main interpretation pitfalls and their use for teaching. Eur. Radiol..

[28] Chen Y, Jiang Z, Guan X (2022). The value of multi-parameter diffusion and perfusion magnetic resonance imaging for evaluating epithelial-mesenchymal transition in rectal cancer. Eur. J. Radiol..

[29] Shen FU, Lu J, Chen L, Wang Z, Chen Y (2016). Diagnostic value of dynamic contrast-enhanced magnetic resonance imaging in rectal cancer and its correlation with tumor differentiation. Mol. Clin. Oncol..

[30] Igarashi T, Furube H, Ashida H, Ojiri H (2018). Breast MRI for prediction of lymphovascular invasion in breast cancer patients with clinically negative axillary lymph nodes. Eur. J. Radiol..

[31] Peng Y, Li Z, Tang H (2018). Comparison of reduced field-of-view diffusion-weighted imaging (DWI) and conventional DWI techniques in the assessment of rectal carcinoma at 3.0T: Image quality and histological T staging. J. Magn. Reson. Imaging.

[32] Youden WJ (1950). Index for rating diagnostic tests. Cancer.

[33] Sedgwick P (2014). Multiple hypothesis testing and Bonferroni's correction. BMJ Clin. Res. Ed..

[34] Mori N, Mugikura S, Takasawa C (2016). Peritumoral apparent diffusion coefficients for prediction of lymphovascular invasion in clinically node-negative invasive breast cancer. Eur. Radiol..

[35] Wang Y, Chen X, Pu H (2022). Roles of DWI and T2-weighted MRI volumetry in the evaluation of lymph node metastasis and lymphovascular invasion of stage IB-IIA cervical cancer. Clin. Radiol..

[36] Schoppmann SF, Bayer G, Aumayr K (2004). Prognostic value of lymphangiogenesis and lymphovascular invasion in invasive breast cancer. Ann. Surg..

[37] Fan WX, Chen XF, Cheng FY (2018). Retrospective analysis of the utility of multiparametric MRI for differentiating between benign and malignant breast lesions in women in China. Medicine.

[38] Zhang S, Zhang D, Yi S (2017). The relationship of lymphatic vessel density, lymphovascular invasion, and lymph node metastasis in breast cancer: A systematic review and meta-analysis. Oncotarget.

[39] Delli Pizzi A, Mastrodicasa D, Marchioni M (2021). Bladder cancer: Do we need contrast injection for MRI assessment of muscle invasion? A prospective multi-reader VI-RADS approach. Eur. Radiol..

[40] Nerad E, Delli Pizzi A, Lambregts DMJ (2019). The Apparent Diffusion Coefficient (ADC) is a useful biomarker in predicting metastatic colon cancer using the ADC-value of the primary tumor. PLoS ONE.

[41] Dijkhoff RAP, Maas M, Martens MH (2017). Correlation between quantitative and semiquantitative parameters in DCE-MRI with a blood pool agent in rectal cancer: Can semiquantitative parameters be used as a surrogate for quantitative parameters?. Abdom. Radiol. (N. Y.).

[42] Hotker AM, Tarlinton L, Mazaheri Y (2016). Multiparametric MRI in the assessment of response of rectal cancer to neoadjuvant chemoradiotherapy: A comparison of morphological, volumetric and functional MRI parameters. Eur. Radiol..

[43] Zhang XM, Yu D, Zhang HL (2008). 3D dynamic contrast-enhanced MRI of rectal carcinoma at 3T: Correlation with microvascular density and vascular endothelial growth factor markers of tumor angiogenesis. J. Magn. Reson. Imaging.

[44] Chen L, Zeng X, Ji B (2020). Improving dynamic contrast-enhanced MRI of the lung using motion-weighted sparse reconstruction: Initial experiences in patients. Magn. Reson. Imaging.

[45] Ao W, Zhang X, Yao X, Zhu X, Deng S, Feng J (2022). Preoperative prediction of extramural venous invasion in rectal cancer by dynamic contrast-enhanced and diffusion weighted MRI: A preliminary study. BMC Med. Imaging.

[46] Michaely HJ, Sourbron SP, Buettner C, Lodemann KP, Reiser MF, Schoenberg SO (2008). Temporal constraints in renal perfusion imaging with a 2-compartment model. Invest. Radiol..

[47] Othman AE, Falkner F, Weiss J (2016). Effect of temporal resolution on diagnostic performance of dynamic contrast-enhanced magnetic resonance imaging of the prostate. Invest. Radiol..

[48] Winkel DJ, Heye TJ, Benz MR (2019). Compressed sensing radial sampling MRI of prostate perfusion: Utility for detection of prostate cancer. Radiology.

[49] Oberholzer K, Menig M, Pohlmann A (2013). Rectal cancer: Assessment of response to neoadjuvant chemoradiation by dynamic contrast-enhanced MRI. J. Magn. Reson. Imaging.

